# The Age of the 20 Meter Solo River Terrace, Java, Indonesia and the Survival of *Homo erectus* in Asia

**DOI:** 10.1371/journal.pone.0021562

**Published:** 2011-06-29

**Authors:** Etty Indriati, Carl C. Swisher, Christopher Lepre, Rhonda L. Quinn, Rusyad A. Suriyanto, Agus T. Hascaryo, Rainer Grün, Craig S. Feibel, Briana L. Pobiner, Maxime Aubert, Wendy Lees, Susan C. Antón

**Affiliations:** 1 Laboratory of Bio and Paleoanthropology, Faculty of Medicine, Gadjah Mada University, Yogyakarta, Indonesia; 2 Department of Earth and Planetary Sciences, Rutgers University, Piscataway, New Jersey, United States of America; 3 Lamont Doherty Earth Observatory Palisades, Columbia University, New York, New York, United States of America; 4 Department of Anthropology, Loyola University of Chicago, Chicago, Illinois, United States of America; 5 Research School of Earth Sciences, The Australian National University, Canberra, Australia; 6 Human Origins Program, National Museum of Natural History, Smithsonian Institution, Washington, D. C., United States of America; 7 Center for the Study of Human Origins, Department of Anthropology, New York University, New York, New York, United States of America; Illinois State University, United States of America

## Abstract

*Homo erectus* was the first human lineage to disperse widely throughout the Old World, the only hominin in Asia through much of the Pleistocene, and was likely ancestral to *H. sapiens*. The demise of this taxon remains obscure because of uncertainties regarding the geological age of its youngest populations. In 1996, some of us co-published electron spin resonance (ESR) and uranium series (U-series) results indicating an age as young as 35–50 ka for the late *H. erectus* sites of Ngandong and Sambungmacan and the faunal site of Jigar (Indonesia). If correct, these ages favor an African origin for recent humans who would overlap with *H. erectus* in time and space. Here, we report ^40^Ar/^39^Ar incremental heating analyses and new ESR/U-series age estimates from the “20 m terrace" at Ngandong and Jigar. Both data sets are internally consistent and provide no evidence for reworking, yet they are inconsistent with one another. The ^40^Ar/^39^Ar analyses give an average age of 546±12 ka (sd±5 se) for both sites, the first reliable radiometric indications of a middle Pleistocene component for the terrace. Given the technical accuracy and consistency of the analyses, the argon ages represent either the actual age or the maximum age for the terrace and are significantly older than previous estimates. Most of the ESR/U-series results are older as well, but the oldest that meets all modeling criteria is 143 ka+20/−17. Most samples indicated leaching of uranium and likely represent either the actual or the minimum age of the terrace. Given known sources of error, the U-series results could be consistent with a middle Pleistocene age. However, the ESR and ^40^Ar/^39^Ar ages preclude one another. Regardless, the age of the sites and hominins is at least bracketed between these estimates and is older than currently accepted.

## Introduction

**Figure 1:**
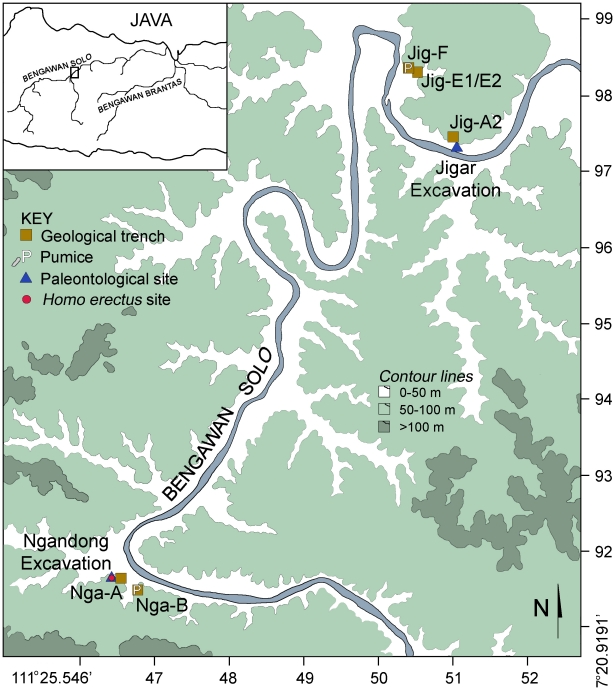
Map of Java showing location of study area (boxed) adjacent to Solo River and a contour map of Ngandong and Jigar localities (base map and latitude/longitude coordinates after J.P. U.S. Army Map Service topographic square 63-046).

**Figure 2:**
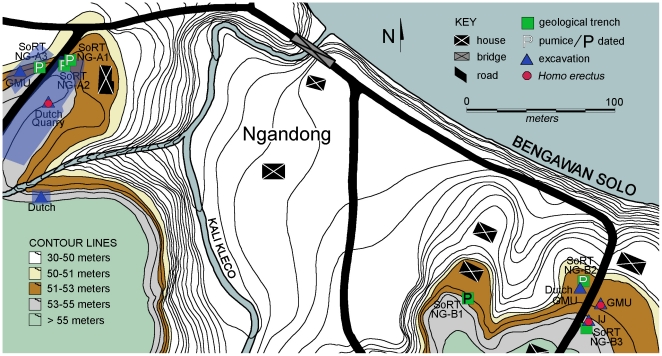
Contour map of Ngandong (modified from [**38**]) with locations of previous excavations [Dutch, Gadjah Mada University (GMU), and Indonesian-Japanese (IJ)], geological trenches (squares) and sample locations of pumice samples (P; this study).

Hominin fossils recovered from sites along the Solo River, Central Java, Indonesia, are widely acknowledged as the youngest representatives of *Homo erectus* [1], [2] (Fig. 1). In the 1930s, excavations in terrace deposits situated 20 meters above the present day Solo River at Ngandong recovered portions of more than 15 individuals along with over 25,000 mammalian fossil specimens. Most of the *H. erectus* fossils are from a less than 1 m thick interval in the lower part of the 20 meter terrace deposits in four laterally adjacent excavation sites (Fig. 2). Faunal teeth collected during these excavations, those in the 1970s, and during our own collections at Ngandong, Sambungmacan, and Jigar yielded ESR and U-series ages consistently younger than 100 ka and suggested that the Ngandong hominins and fauna may be as young as 35–50 ka [3]. Such ages implied that *H. erectus* survived into the late Pleistocene in island southeast Asia and was a contemporary of *H. sapiens* [3], [4]. Yet, the ages are surprisingly recent given that the last appearance datum (LAD) of *H. erectus* in Africa and the rest of Indonesia is about 1 Ma [5], [6], with a LAD of perhaps 300 ka or even earlier in China [7], [8]. While the geomorphology of the Solo River terrace and faunal correlations [9], [10] suggested an early late Pleistocene age for Ngandong, the morphology of the hominins and the presence of certain mammalian taxa (e.g., *Stegodon*) had been broadly viewed as more consistent with a middle Pleistocene age [11], [12].

Thus, the young ages were met with some skepticism [13], despite being internally reproducible, congruent with prior U-series ages on bones from Ngandong [14]–[16], and supported by similar ages on mammal teeth from the *H. erectus* site of Sambungmacan and the faunal site of Jigar [3]. This skepticism rejuvenated the argument that the hominins might have been selectively reworked from older sediments [13], [17]–[19] and/or that these particular ESR and U-series ages were inaccurate and too young [13], [20]. Although the Ngandong deposits are clearly fluvial in origin, suggesting that reworking could play a role in assemblage formation, the recent, direct γ spectrometric ^230^Th/^234^U age determinations on the hominins themselves (Ngandong 1 and 7, and Sambungmacan 1) that yielded ages bracketed between 40 and 70 ka [4], appear to discredit the selective reworking hypothesis. The stratigraphy and sedimentology in our excavations and trenches at Jigar and Ngandong (see Appendix S1), similarities of fluorine content and fluorine/phosphate ratios between Ngandong 5 and a hippo from the site [21], and recent taphonomic analyses of the fauna and hominins (see Appendix S2) also argue against either multiple episodes of deposition or the selective reworking of only the hominins (or only the fauna). Similarly, archival work by other researchers argues for a single depositional event of relatively short duration at least for the Ngandong hominins and fauna [22].

## Methods

The objective of our Solo River Terrace project (SoRT) is to understand the site formation processes of these sedimentary deposits in order to clarify the age of the hominins and fauna. To this end, we have undertaken excavation, survey, and geotrenching since 2004 and have obtained ^40^Ar/^39^Ar ages on water lain pumices found interbedded within the fossiliferous deposits at Ngandong and Jigar and ESR and U-series ages for fauna recovered during our Jigar excavations (Figs. 2–5). The sedimentology methods and results and details of the taphonomic analyses are reported in Appendices S1, and S2, respectively.

**Figure 3 pone-0021562-g003:**
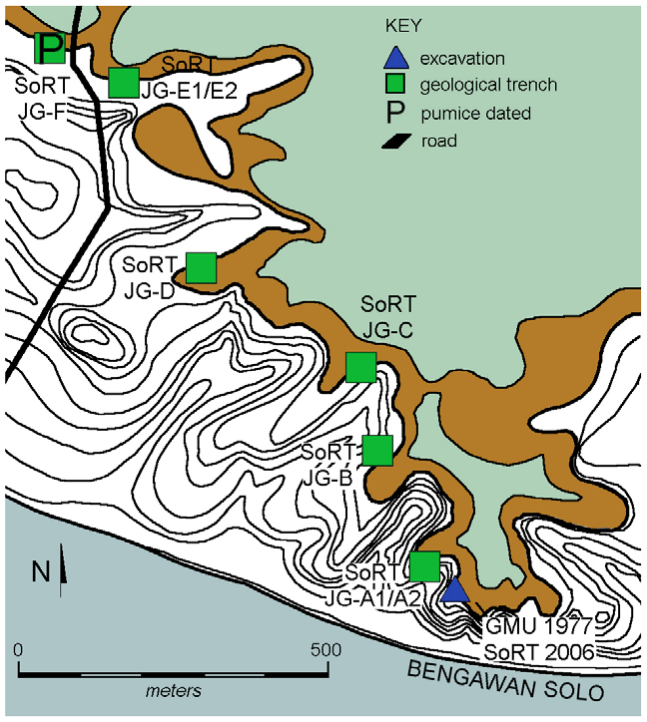
Contour map of Jigar (base map and latitude/longitude coordinates after J.P. U.S. Army Map Service topographic square 63-046) with locations of Gadjah Mada University (GMU) and this study (SoRT) excavations (triangle), geological trenches (squares) and sample locations of pumice samples (P; this study).

**Figure 4 pone-0021562-g004:**
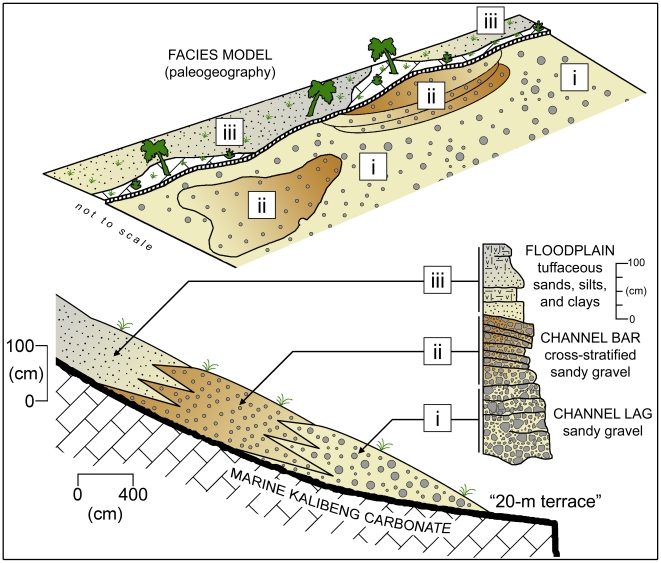
Generalized composite sedimentary geology of the Ngandong and Jigar “20 meter terrace" denoting stratigraphic positions of *Homo erectus* fossil material and dated pumices. At Ngandong, especially in the area in the vicinity of the GMU and Dutch Ingraving 2 excavations, the original erosional basal channel morphology of the 20 meter terrace cut into the Kalibeng is well exposed showing a gradual rise away from the modern Solo River after ascending abruptly from the present river level. Moreover, each of the components of the fluvial association is placed at discrete altimetric positions relative to the water level of the modern river with facies changes at defined distances lateral to the channel (Fig. 5). In our excavations and geotrenches at Ngandong and Jigar, vertebrate remains occur primarily in the basal lag and bar deposits; that is in the lower meter of a 3 to 5 m veneer of fluvial sediments overlying the erosional bench cut into the marine Kalibeng Formation.

**Figure 5:**
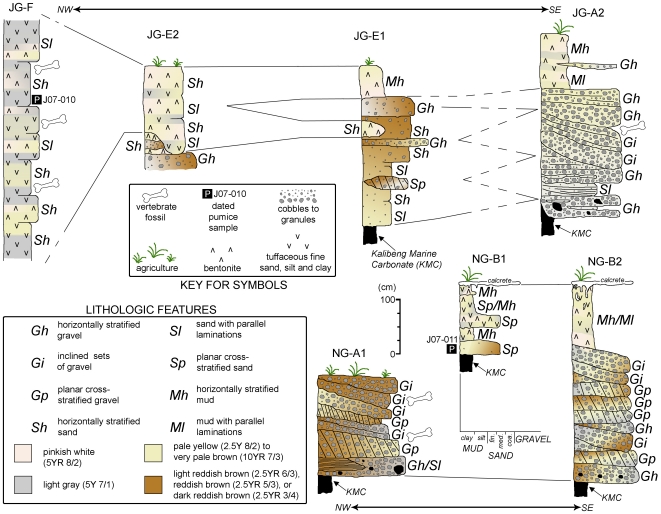
Sample stratigraphic sections from Jigar (JG) and Ngandong (NG) trenches and excavations. JG sections, NG-A1 and NG-B1 this study, NG-B2 after GMU excavations. JG-A2 is adjacent to the 2006 Jigar excavations, the ESR and U-series samples come from the equivalent of these gravels. The stratigraphy is discussed in greater detail in the Supplemental Information in reference to this figure.

### ^40^Ar/^39^Ar analyses

^40^Ar/^39^Ar measurements were undertaken at the Noble Gas Laboratory of Rutgers University using methods similar to those described in detail by us in Carr et al. [23] and again in Turrin et al. [24]. Pumices for dating were selected that showed the least amount of weathering or alteration, rounding or abrasion due to fluvial transport. The pumices were cleaned of any attached matrix, gently crushed by hand using a mortar and pestle, sieved to 20 to 40 mesh size range, washed in an ultrasonic bath to remove any remaining fines and attached matrix, and dried in an oven at approximately 80°C. Using a binocular microscope, euhedral hornblende crystals were then hand-picked using fine pointed tweezers. Plagioclase in these pumices are typically of extremely low K (less than 0.2% K_2_O), show excess or trapped Ar components, and have proven to be of minimal use in dating the Central Java pumices. The hornblende was then loaded into sample pits in aluminum irradiation disks along with interspersed sample pits of the monitor mineral Alder Creek (AC-1) for the calculation of the irradiation parameter *J*, and to monitor any irradiation flux gradient. The loaded aluminum sample disks were then wrapped in aluminum foil and encapsulated in a quartz tube. The encapsulated samples were irradiated for 10 minutes in the Cadmium-lined, In-Core Irradiation Tube (CLICIT) facility at the Oregon State University TRIGA Research Reactor.

Following irradiation, approximately 100 mg of irradiated hornblende was equally split and loaded into round, six mm diameter sample wells in a 60 mm stainless steel sample disk. To account for any irradiation gradient, three crystals of the monitor mineral AC-1 were loaded into eight two mm diameter pits for each of six monitor mineral positions interspersed amongst the irradiated samples. The sample disk was then loaded into the sample chamber, sealed, evacuated, and baked overnight at 100°C.

Incremental heating of the hornblende was accomplished using a 40-watt CO_2_ laser passing through a faceted lens that produces a six mm square beam. The power gradient across this beam is relatively “flat" showing little variation in the temperature profile over the heating area. During heating, the laser is “jogged" in a diamond pattern at a uniform rate to ensure that the six mm diameter round sample pit is evenly heated.

Samples were heated incrementally via step-wise increases in laser wattage output, typically 10 steps, from approximately 400°C to 1400°C, or until the samples were fused into glass balls and minimal Ar was measurable above background values. Argon isotopes were measured on a MAP-215-50 mass spectrometer using a Blazers multiplier operating in pulse counting mode. Mass discrimination was monitored during the analysis period by measuring interspersed aliquots of air delivered from an automated air pipette system. The mass discrimination values were then plotted against time, linearly fit, and the resultant time averaged mass discrimination was then applied to the unknowns [24]. In three of the experiments (sample 30655, 30656 and 30657), samples were laser heated incrementally in step-wise fashion to 12 watts and were then heated at higher increments until fusion at a later time following low temperature degassing of all samples. This procedure was used to minimize the affect of increased background memory as a result of the release of relatively high atmospheric ^40^Ar during the low temperature heating. Applied discriminations are those measured and fit during each heating phase (Table S1). Data collection and data reduction were performed using the software “MassSpec" by Alan Deino. Typical extraction line blanks for the Rutgers University system at the mass-to- charge ratios of the pertinent Ar isotopes are: M/e 40 = 3×10^−16^ mol, M/e 39 = 4×10^−19^ mol, M/e 38 = 8×10^−19^ mol, M/e 37 = 2×10^−17^ mol, and M/e 36 = 4×10^−18^ mol.

**Figure 6:**
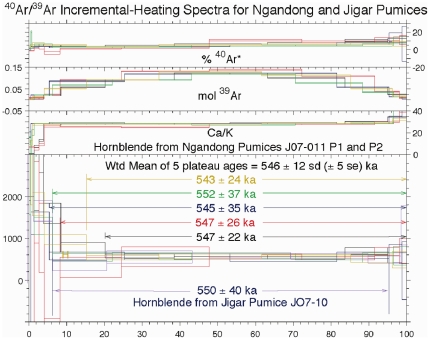
Incremental heating spectra for Ngandong (upper graphs) and Jigar (lowest graph) pumices.

The neutron fluence parameter, “*J*," for the sample irradiation was determined using an age of 1.194±0.014 Ma for sanidine from the rhyolite of Alder Creek, California [25]. *J* = 7.04±.03×10^−5^. Interfering neutron reactions from Ca and K were corrected using the following values (^36^Ar/^37^Ar)Ca = (2.72±0.06)×10^−4^, (^39^Ar/^37^Ar)Ca = 7.11±0.02)×10^−4^ [25], [26]. Age calculations were made using the currently accepted decay constants and isotopic abundances [27]: λ_ε_ = 0.581×10^−11^,yr^−1^, λ_β−_ = 4.962×10^−10^ yr^−1^, ^40^K/K_total_ = 1.167×10^−4^. Summary results are reported in Figure 6 and the full data set in Table S1.

### ESR and U-series analyses

As age control was an important component of our research strategy, during our 2006 excavations in-situ faunal teeth were identified to be used for ESR and U-series analyses. As suitable teeth were exposed during excavation, five sediment samples (four from around the sides of the tooth and one below the tooth) were collected for dose rate calculations. Relative stratigraphic position to one another and depth from surface was known for all samples. All samples came from two adjacent 1×1 m excavation units that lie a meter or two beneath the position of the pumices dated from Jigar; however, these fossils dated by U-Series and ESR derive from sediments that also locally interbed with the volcanoclastics bearing the dated pumices.

The procedures for U-series and ESR analysis followed those routinely applied in the Australian National University ESR dating laboratory. From each tooth, an enamel fragment with attached dentine was removed and analyzed. For uranium elemental concentrations as well as U-series isotope ratios laser ablation ICP-MS [28], [29] was used on a thin slice located between the two subsamples. U-series values could only be obtained on the dentine. The sediments were analyzed for U, Th, and K by solution ICP-MS (Genalysis, Perth). For ESR dose analysis, the enamel was powdered and successively irradiated in 11 steps to 578 Gy. Radiation doses were monitored with alanine dosimeters and evaluated against a calibrated dosimeter set (A. Wieser, Messtechnik, München). Dose values were obtained by fitting the natural spectrum back into the irradiated ones [30].

Chemical data were converted into dose rates using conversion tables [31]. For the calculation of the internal dose rate values, Monte Carlo beta attenuation values [32] and an alpha efficiency of 0.13±0.02 [33] were used. The external beta and gamma dose rates were derived from the chemical analysis of the sediment samples which surrounded the teeth. All analyses assumed a water content of 20±10%. The cosmic dose rate was calculated from the present day sample position [34], [35]. For comparison with earlier results [3], ESR ages were calculated for closed system (EU: early U-uptake) and linear U-uptake (LU). Where possible, U-series and ESR data were combined for open system modelling, assuming that the U-series ratios in the enamel were the same as in the dentine [36]. Age calculations were carried out with the ESR-DATA program [37]. Summary results are reported in Table 1 and the full data set in Table S2.

**Table 1:** Summary Results for ESR and U-series for Jigar excavations in 2006^a^.

Lab. No	cmbs	De	De-error	U-series	error	EU(ka)	error	LU(ka)	error	US-ESR	+-error	-error
		Gy	Gy	ka	ka	ka	ka	ka	ka	ka	ka	ka
2372A	79	74.6	1.8	63.7	0.1	46	2	68	4	MV		
2372B		87.6	3.1	63.7	0.1	50	3	75	5	MV		
2375A	82	138.2	8.0	173.8	0.4	57	4	90	7	leach		
2375B		135.5	5.1	173.8	0.4	56	3	88	5	leach		
2369A	86	102.5	3.6	68.3	0.5	67	4	97	7	MV		
2369B		111.9	4.8	68.3	0.5	72	5	105	8	77	10	8
2377A	93	97.0	3.0	285.3	2.9	62	3	92	5	leach		
2377B		92.4	3.0	285.4	2.9	61	3	91	5	leach		
2373A	95	86.2	2.5	129.7	2.1	59	3	86	5	leach		
2373B		98.3	2.6	129.7	2.1	64	4	94	6	leach		
2376A	138	80.5	2.3	157.8	0.4	54	3	77	5	leach		
2376B		83.1	3.8	157.8	0.4	58	3	82	5	leach		
2370A	143	82.9	2.3	68.5	0.5	47	2	70	4	MV		
2370B		75.5	4.2	68.5	0.5	44	2	66	4	MV		
2368A	168	172.2	6.1	82	1.1	92	6	139	10	105	14	12
2368B		207.1	8.6	82	1.1	107	8	163	13	143	20	17

a De = Dose; EU: Early U-uptake (closed system); LU: Linear U-uptake; US-ESR: combined U-series/ESR age estimate [36], MV = Model violations (closed system U-series between EU and LU ESR ages), Leach: U-series age ≫LU (ESR); For the full data set, underlying assumptions, and model calculation references see Table S2.

## Results

The deposits of the 20-m terrace exposed at Jigar and Ngandong consist of poorly to moderately sorted assemblages of terrigenous clastics (see Appendix S1). The 20-m terrace sedimentary package comprises a vertical facies association having (i) sets of horizontally stratified sandy to pebbly gravel, (ii) planar cross-stratified sets of sand and sandy gravel interbedded with inclined sets of sand and sandy gravel, and (iii) horizontally stratified sets of tuffaceous silts, sands, and clays, that may preserve small-scale planar/trough cross-stratifications, ripple cross-laminae, parallel laminae, and carbonate nodules. At Ngandong, the basal pebbly gravel is relatively thin and discontinuous, whereas at Jigar the pebble gravels appear better developed, thicker, and more continuous (Figs. 4, 5; Additional geological and taphonomic results can be found in Appendices S1 and S2). At Ngandong, the sequence is capped locally by a calcrete.

Accumulation of the sedimentary package is interpreted to be of limited geological duration, probably accumulating with subsiding flow following high or flood stage intervals. The basal pebbly gravels, primary sedimentary structures associated with the sands and gravels, and general fining-upward nature of the sediments are interpreted as a fining upward fluvial succession associated with channel and overbank areas of a river system. Basal contacts with the marine Kalibeng Formation are typified by erosional surfaces, overlain by andesitic pebbly gravels that also appear to be channel lag. Ample cross-stratification in the overlying unit is indicative of channel bar deposition. The upper, tuffaceous, fine grained strata, with secondary pedogenic carbonate features, suggest intermittent low energy flooding as a result of waning current flow, or floodplain deposition some distance from the active channel(s). Since these fines intercalate with and directly overlie channel-bar strata they may record within-channel deposition during the final stages of infilling/abandonment. We record at least three fining-upward intervals within the middle and upper units, but the entire package appears to be a single storied sequence and most likely represents only the last phase of fluvial deposition of the ancient river system leaving the deposits preserved on a terrace bench some 20 meters above the present day level of the Solo River. This fluvial association and its components are more sporadically exposed and persist laterally for meters to tens of meters at Jigar, but are traceable fairly continuously over hundreds of meters along the 20 meter terrace at Ngandong (Figs. 4, 5; see Appendix S1 and included figures).

During recent field examination, we discovered both water lain tuffs and surprisingly large pumices in the same stratigraphic levels as had produced the earlier hominins and the dated bovid teeth. Smaller rounded pumices and well rounded andesite pebbles occur in the basal lag; however larger, up to 15 cm size, better preserved pumices come from the middle cross-bedded or bar deposits intermixed with fossil vertebrate remains at numerous sites at Ngandong and Jigar. No pumices or pure volcanic ashes were found in the uppermost fine-grained deposits at Ngandong, but some were found in association with fossil vertebrate remains in the finer-grained tuffaceous silts and sands at Jigar. The pumices are not in large concentration, but appear to be of similar composition, and widely dispersed within this interval along the 20 meter terrace. We previously reported small (mm size) pumices from the fossiliferous layers at Ngandong; however, these yielded insufficient gas for accurate ^40^Ar/^39^Ar age determination [3]. Pumices from Jigar occur within and above fossil bearing strata including our 2006 excavation levels from which the ESR and U-series ages result (Fig. 3) and the 1970s excavations [38], as well as from levels in which bovid teeth were collected for the 1996 ESR and U-series dating study [3]. The pumices at Ngandong are from the hominin bearing levels of the excavations by Gadjah Mada University (GMU) in the 1970s and 1980s [39], and the second Dutch excavation (2^nd^ Ingraving) of the 1930s [10], (Fig. 2). Additional pumices come from several areas of the cross-bedded bar deposits in undisturbed strata preserved in remnants of the Dutch Ingraving I site that yielded the hominins in the 1930s (Fig. 2). Bovid teeth from these GMU excavations also yielded late Pleistocene ages similar to the γ spectrometric ^230^Th/^234^U ages for two of the hominin calvariae from the first Dutch excavation (1^st^ Ingraving) of the 1930s.

^40^Ar/^39^Ar incremental heating analyses on hornblende separated from these pumices appear internally reproducible and of limited variability; they yield an average age of 546±12 ka for both sites (SD, 5 SE; Fig. 6; Table S1). Plotting the data for the plateau steps derived from the five incremental heating experiments of JO7-011 P1 and P2 from Ngandong on an inverse correlation diagram, the 28 data points form a well-behaved linear mixing line with an atmospheric ^40^Ar/^36^Ar intercept of 295.2±0.8, indistinguishable from an expected value of 295.5. The fit of the data indicates an isochron age of 557±3 ka, an age within error of the plateau age. The MSWD (essentially the ratio of the scatter about the line fit relative to the measurement error in which a value of one suggests that the scatter in the isochron line fit is due to measurement error) of 0.36 suggests over estimation of the reported measurement uncertainties. Overall this is a well behaved hence reliable age. Two incremental heating analyses were made on hornblende from a pumice from Jigar (JO7-10), the second of which (Table S1, RUID 30665) resulted in a plateau age of 550±40 ka. For J07-10, the low intermediate temperature steps showed a high degree of scatter possibly reflecting the greater degree of weathering noted for this pumice. If we omit the data from these increments, and plot the remaining 5 of 9 data points, we obtain an ^40^Ar/^36^Ar intercept of 295.5±1.5, an MSWD of 0.88, and an isochron age of 551±4 ka, an age within the error of the plateau age.

The pumices are thus of middle Pleistocene age. Such an age is significantly older than the ESR, U-series, and γ spectrometric ^230^Th/^234^U age ages on fossil bone and teeth [3], [4], and significantly younger than any reported ^40^Ar/^39^Ar ages on pumice or tuff associated with earlier *H. erectus* fossils from the Sangiran and Bapang formations of Sangiran, Java [40], [41]. The ages are similar, however, to ages of 539±18 ka and 571±16 ka (BPN13 and BPN15) that we obtained on hornblende bearing pumices from the non-hominin-bearing Pohjajar Formation at the top of the Sangiran sequence (see Table S1). Notably, we did not obtain any ages similar to those reported for the hominin bearing pumiceous deposits (Grenzbank or Bapang formations) of the Sangiran Dome region. The age of the Solo pumices suggests a possible correlation between the “20 meter terrace" fauna of Ngandong and Jigar and the poorly known “Notopuro" fauna (although the type Notopuro is significantly older than the Pohjajar Fm), a correlation that requires further investigation.

On the other hand, the closed system (EU) ESR age estimates range between about 44 and 100 ka (Table 1). They are older than the previously published results [3]. For open air sites it is virtually impossible to speculate which parametric U-uptake model may yield the correct age estimate [42]. Further insights are given by the additional U-series analyses (Table 1 and Table S2). Samples 2373, 2375, 2376, and 2377 yielded significantly older U-series results in the dentine than indicated by the EU ESR result. In these cases it has to be assumed that serious U-leaching has taken place at some time in the past. For samples 2370 and 2372, the U-series results lie between the calculated EU and LU ESR ages, indicating modest leaching. Open system modelling is only possible for samples 2368B and 2369. Both indicate an early late Pleistocene age. It is remarkable that all samples, except the lowermost (2369), have closely similar EU ages (57±8 ka). This is only slightly larger than the reproducibility of the subsamples of one tooth and is consistent with our sedimentological observations during excavation. The ESR data of most of the samples (except 2368) imply that they experienced a very similar dose rate environment and are of similar age, consistent with the view that relatively little reworking has taken place.

Recent detailed analysis on tooth enamel fragments have shown that enamel contains more than one type of CO_2_^−^ radicals that constitute the ESR signal used for dose estimation, some of these may be unstable [43]. Preliminary results on samples from other regions indicate that ESR may underestimate the correct dose value by up to 30% [44]. Such an underestimate would make little difference to the results, except that U-series modelling was possible for sample 2368A. The average EU age estimates of the samples, except 2369, would rise to 72±10 ka and the US-ESR result of 2368 to around 110 ka. The strong discrepancy between the ESR and U-series results of most of the samples can only be explained by U-leaching. It is noteworthy, that the γ spectrometric analysis of the Ngandong and Sambungmacan [4] calvariae also indicated leaching of between 20 and 50%, based on the comparison of Th/U and Pa/U data as did our earlier work at these sites and Jigar [3].

Combined ESR and U-series ages were possible for only three of the specimens. These specimens yield ages of 77 to 143 ka for combined U-series and ESR analyses (69–163 ka for the lower and upper ranges of individual ESR or U-series analyses for the same specimens). Regardless of the vagaries of the mobilization of U-series isotopes as well as dose estimation, these samples indicate a late Pleistocene age. The lowermost sample may be as old as 200 ka.

## Discussion

In light of these results we are faced with a conundrum of opposing, yet internally consistent, estimates from different dating systems. This raises two possible scenarios: the pumices may all be reworked from older deposits, such as the Pohjajar Formation, and do not reflect the age of the Solo River deposits or hominins; alternatively, the ESR, U-series, and γ spectrometric ^230^Th/^234^U age determinations on teeth and bones, although internally consistent, may not be dating the age of deposition but of some other events of more recent hydrological activity associated with U-mobilization (U-uptake as well as leaching), [42]. While the exact nature of such events are unknown, they may record a change in paleoenvironmental context, change in the hydrology of the 20 meter terrace, or changes in groundwater movement or percolation following change in Solo River levels.

Although the pumices are waterlain there is little to support the idea that they are reworked. The large size and freshness of the pumices and their context intermixed with fossil vertebrate remains with little evidence of rounding suggests rapid accumulation of both fossils and pumices in channel bar deposits that formed over a geologically short time scale. Because pumices are friable and easily broken, large pumices are not easily reworked over great intervals of time or space and are preserved only if rapidly buried (see Appendix S1). Other currently known middle Pleistocene sediments of appropriate age are minimally 30 km away, which would preclude them as a source from which these large pumices could have been reworked; however, the presence of other more locally available but currently unknown or unpreserved sources cannot be excluded. Additionally, dating of pumices in Sangiran and elsewhere on Java, has shown that water lain pumices, when stratigraphically limited in context, yield ages consistent with primary interbedded tuffs [40], [41]. Thus, we consider it more likely that pumice deposition occurred shortly after or geologically contemporaneously with their volcanic eruption and deposition into the paleo Solo River. Others have made a similar argument for the short duration of deposition of the Ngandong hominins and fauna [22]. Regardless, for the first time these pumices yield firm radiometric evidence of a middle Pleistocene contribution to the 20 meter terrace and certainly provide a maximum age for the sites.

The present ESR and U-series results are somewhat older than the previous set from Jigar, but also show evidence of uranium leaching. In most instances, closed system ESR and U-series are likely to be either actual or minimum ages. A compilation of U-series results from open air sites, shows that apparent closed system U-series results on dental tissues (enamel, dentine, cement) may underestimate the likely age of the site by up to a factor of 20 [42]. As such, the U-series data measured on the Jigar faunal material could be expected from a site with an age in the 500 ka region.

The problem arises from the ESR results. Over 500 ka, the present day external beta, gamma, and cosmic dose rates would generate a dose value of around 398±15 Gy (Table 1). This is an absolute minimum value that the teeth would have received as any uranium dose rate contributions from the dental tissues are ignored. Only one sample, 2368, comes close to this minimum dose value but would still underestimate a 500 ka age by more than 35%. The other samples would underestimate by between 55 and 75%. Any U-uptake, regardless of how it happened in the past, would generate additional dose contributions. Using the present day U-concentrations and U-series isotope values the doses required to reconcile a 500 ka age would range between 400 and 1100 Gy. Sample 1369 's dose value is around 40% of the 500 ka target dose, while the others present only 17±6%. While it may well be possible that systematic ESR age underestimations in the 30% range may occur [44], there is so far little evidence that ESR underestimates middle Pleistocene ages by a factor of six. While isotope mobilization and fluctuating water contents in the sediments could be responsible for some changes in the past external dose rate, we are not aware of any published or unpublished results that could explain the large offsets with the ESR results. On the other hand, it may well be that teeth from humid, tropical regions behave differently than those that revealed a potential 30% age underestimation [44]. This clearly requires further, detailed investigation.

Our results raise several issues for the interpretation of the Ngandong site, the intercalibration of ^40^Ar/^39^Ar age estimates and other chronometers, and the evolution of *H. erectus*. On the basis of geomorphology, absence of clear indicators of time-transgression at the site (e.g., mixed deposits), and the reasonable consistency of the ESR and U-series results, which argue against reworking, we suggest that, whatever their age, the fauna and hominins are of similar geological age. In the absence of any reliable ^40^Ar/^39^Ar age estimates, some of us have previously favored this to be a late Pleistocene age [3], [4]. We continue to see no basis for dismissing the earlier open system ESR and U-series dates because they meet the scientific criteria for acceptability applied to these systems. That is, they are nonrandom and internally consistent with the stratigraphy of the sites. At other sites, dates from these systems with similar levels of technical accuracy are considered acceptable [45], [46]. Therefore, without dismissing the systems entirely, we cannot dismiss the dates. It also seems unlikely to us that a suite of systems (ESR, U-series, γ spectrometry) and separate investigations on a variety of different teeth, bones, and taxa from Ngandong and other sites in the Solo River terraces would coincidentally yield the same general ages if they were not dating some actual phenomenon. It is the case that this age may not be that of deposition of the site, and we raise the possibility that these ages date some later geomorphologic or hydrologic event (other than the initial deposition of the sediments and hominins). Nonetheless, these age estimates certainly supply a minimum age for the site.

At the very least, it now seems possible to bracket the age of the deposits at Ngandong and Jigar with a maxima of 546 ka based on the argon results and a minima of 143 ka based on the oldest of our fully modeled combined ESR/U-series ages. It is certainly possible that the age of the hominins more closely approaches one than the other of these extremes, which given the geochemical issues with U-series at this site and the apparent site formation processes, some of us suspect is more likely to be the argon age. Technically, however, the argon age is a maxima for the sites. And, we caution that the dating results of the hominin material [4] agree well with the current ESR results, regardless of whether they are taken at face value, or a systematic age underestimation of 30% is assumed. And these results indicate a late Pleistocene age.

If the middle Pleistocene ^40^Ar/^39^Ar ages better reflect the age of the Solo River 20 meter terrace deposits and hominins, the site of Ngandong remains a relatively late source of *H. erectus*; however, these *H. erectus* would not be the contemporaries of Neandertals and modern humans, and their chronology would widen the gap between the last surviving *H. erectus* and the population from Flores – whose source population has been argued to be Indonesian *H. erectus* [47], [48]; although this point is contested, [49]. Instead, the Ngandong hominins would be contemporaries of the *H. heidelbergensis* from Atapuerca, Spain and elsewhere in Europe, and, possibly the archaic *H. sapiens* specimen from Bodo (Ethiopia), which might favor arguments that they are more closely affiliated with these taxa and differ from *H. erectus* [50], [51]. Such ages for Ngandong would suggest that a series of geographically relatively isolated lineages of hominins lived during the middle Pleistocene.

## Supporting Information

Table S1 Results of incremental heating analyses for ^40^Ar/^39^Ar analyses for Ngandong, Jigar and Pohjajar pumices.
(XLSX)

Table S2 Results for ESR and U-series analyses for Jigar samples.
(XLS)

Appendix S1 Sedimentology, stratigraphy, and implications for the dated samples. 1.1 Description of Sedimentology and Stratigraphy. 1.1.1a Sedimentological overview. 1.1.1b Stratigraphic relations and depositional history of dated volcaniclastic and faunal samples. 1.1.1c References. 1.2 Figure and legend. Figure 1.2.1. Sedimentary features of the “20-m terrace".
(PDF)

Appendix S2 Taphonomy of faunal remains from Jigar. 2.1 Taphonomy: materials, methods, and results. 2.2 Figures and legend for taphonomy. Figure 2.2.1 Maximum bone length. Figure 2.2.2 Surface readability.
(PDF)
