# Supplementary material for: The Age of the 20 Meter Solo River Terrace, Java, Indonesia and the Survival of Homo erectus in Asia
Source: PLoS One. 2011 Jun 29;6(6):e21562. doi: 10.1371/journal.pone.0021562 (PMC3126814; doi:10.1371/journal.pone.0021562)
Supplement: Appendix S1 — Sedimentology, stratigraphy, and implications for the dated samples. 1.1 Description of Sedimentology and Stratigraphy. 1.1.1a Sedimentological overview. 1.1.1b Stratigraphic relations and depositional history of dated volcaniclastic and faunal samples. 1.1.1c References. 1.2 Figure and legend. Figure 1.2.1. Sedimentary features of the “20-m terrace". (PDF) [file pone.0021562.s003.pdf]

Appendix S1. **Stratigraphy, sedimentology, and implications for the dated volcaniclastics**

Supporting Information for The age of the 20 meter Solo River Terrace, Java, Indonesia and the survival of *Homo erectus* in Asia. Etty Indriati, Carl C. Swisher III, Christopher Lepre, Rhonda L. Quinn, Rusyad A. Suriyanto, Agus T. Hascaryo, Rainer Grün, Craig S. Feibel, Briana L. Pobiner, Maxime Aubert, Wendy Lees, Susan C. Antón

S1.1 Description of Sedimentology and Stratigraphy

Members of the Solo River Terrace (SoRT) project undertook archaeological excavations, geological trenching, and survey in Java during 2004, 2006, 2007, and 2008. Geological sections from the Dutch excavations of the 1930s and the GMU excavations of the 1970s and 1980s also inform our stratigraphic discussions. Here we report the geological results relevant to understanding the implications of the dated samples. In particular, we address the key issue involved with dating the Ngandong and Jigar fossils: the time lag between the depositional age and the eruptive age of the dated pumices.

S1.1.1a Sedimentological overview (Figure S1.2.1)

Fluvial deposits at Jigar and Ngandong include gravel and coarser sand, in addition to tuffaceous silt, finer grained sand, and some clay. The fine-grained deposits overlie and have interbedded relationships with the coarser ones; the contact between the two is gradational or abrupt. Basal contacts for the gravels and coarser sands are invariably erosive and most often with Kalibeng marine carbonates. As a consequence, cobble-sized and angular rip-up clasts of marine carbonate are incorporated into the lowermost gravel/sand layers. Typically there is irregular erosive relief developed on the topmost portions of the Kalibeng (Fig. 3 of main text).

Primary sedimentary structures are an important key to the interpretation of depositional setting for the Jigar and Ngandong sequences. Planar cross-stratification is particularly well developed throughout the gravels and coarser sands. Individual cross-stratified sets have thicknesses from about ten centimeters to a few decimeters, and exposed lateral extents from ten centimeters to a meter or two. Larger-scale planar cross-stratified sets occur as cosets with composite sets separated by a subtly erosive and slightly concave-up surfaces. Contrastingly, smaller-scale planar cross-stratified sets can be either an isolated occurrence surrounded by relatively structureless sediments or a stack of multiple sets. Stacked cross-strata are orientated in various directions; however, in several instances the orientation changes are systematic as they successively alternate in opposing directions. Horizontally stratified sets are observed for the fine-grained deposits and for the gravels and coarser sands. Individual sets are decimeters to a meter thick and have nearly planar or undulatory upper and lower boundaries. Small-scale cross-strata, ripple cross-laminations, parallel laminations, and tabular/nodular carbonate concretions are locally observable in the sets of fine-grained strata.

Primary sedimentary structures, grain-size distributions, and stratigraphic relationships suggest the deposits are of fluvial origin, with the gravel and sand representing within-channel settings and the tuffaceous silt, finer sand and clay indicating the fine-grained infilling of channel segments in addition to overbank areas (e.g., ref. 1). Planar cross-stratification indicates two-dimensional dunes and channel bars with

slipfaces (2). Small-scale cross-strata with successively alternating orientations may indicate short-time current changes resulting from, for example, reversing flows due to tidal processes that affect rivers in near-coastal settings (3). Horizontally stratified sets of sediment suggest upper plane-bed deposition and the rapid fallout of grains from a detrital-laden, short-lived flow (4). The clast sizes, fabrics, carbonate concretions, and stratigraphic positions relative the coarse clastics suggest the tuffaceous fine-grained strata accumulated in low-energy floodplain settings and/or within channel segments undergoing abandonment (5).

S1.1.1b Stratigraphic relations and depositional history of the dated volcaniclastic and faunal samples (Fig. 3 of main text)

A key issue involved with age of the hominin remains is the stratigraphic association of the dated pumices and faunal samples with the other hominin-bearing clastics. At Ngandong, the observed vertical stratigraphic order is: Kalibeng marine carbonates, followed by fluvial gravels and coarse sands, capped by tuffaceous fine-grained strata. This fundamental order is observed at lithostratigraphic log Ng-B1 near the GMU/IJ excavations and from the hominin cranial site of the GMU excavations, where one of the dated pumice clasts derives (see log Ng-B2). At the hominin-bearing Dutch excavation of Ngandong, the succession is Kalibeng marine carbonate followed by fluvial gravels and sands (log Ng-A1 and -A2). The ESR/U-series dated mammalian tooth samples (6) were collected from the fluvial gravel sands.

A similar stratigraphic order of Kalibeng marine carbonates, fluvial gravels and coarse sands, and tuffaceous fine-grained sediments is also apparent at Jigar (e.g., log Jg-A2 and 2006 Jigar excavation). The ESR dated mammalian tooth samples (this study) collected from two of the 2006 Jigar excavation squares all derive from the gravel and coarse sand layers. A different situation, however, is illustrated by log Jg-E1 that shows decimeter-thick lenses of tuffaceous fine-grained strata interbedded with the gravels/sands. Locally, across <5 m of exposure, these lenses expand vertically and form a ca. 2-m-thick tuffaceous fine-grained succession with little intercalated gravel/sand (log Jg-E2). This succession can be traced laterally for another 10 m, in the direction of the modern Solo River, where it further expands to over 5 m and has a lateral extent measuring some 30 m. The Jigar dated pumices are from this expanded outcrop (see log Jg-F).

These stratigraphic aspects combined with the sedimentology suggest all of the fluvial sediments were accumulated from a series of genetically related depositional events occurring at either one location or separate penecontemporaneous locations of the same Pleistocene river system. Besides the good preservation and large clast sizes of the dated pumices, both stratigraphic and sedimentological evidence suggest the volcaniclastics and fluvial gravels and sands were deposited fairly rapidly, shortly after eruption. Aforementioned stratigraphic relationships between the tuffaceous fine-grained strata and gravels/sands at Jg-E1, -E2 and -F resemble the interstratifications formed when volcaniclastics are injected into fluvial channels carrying a mainly terrigenous load. Mack et al. (7) describe analogous situations for pyroclastic eruptions that flooded the ancestral Rio Grande River (U.S.A.) with volcaniclastics that were transported (>400 km) and deposited as layers (<2 m) interbedded with terrigenous detritus. Deposition took

place over days to months according to these authors. Pyroclastic eruption(s), perhaps from Mount Lawu at Central Java, dumped quantities of tephra/pumice into the Pleistocene Solo River—thereby temporarily choking reaches and limiting the transport of gravels/sands as well as the flow of water. Soon after the eruption, however, the blockage was breached and the volcaniclastics moved downstream. At first, with there being little flowing water and ample volcaniclastic debris, the volcaniclastic materials presumably traveled *en masse* as viscous slugs and came to rest quite rapidly as they quickly lost momentum. Such a situation may account for tuffaceous successions at Jigar/Ngandong that lack primary sedimentary structures: viscosity and rapid accumulation for the tuffaceous fine-grained strata prevented the formation of effective hydrological processes and thus structures. On the other hand, the tuffaceous fines with good horizontal stratification and evident structures probably represent "typical" bed- or suspended-load transportation and deposition when the ratio of detritus to water decreased toward pre-eruptive conditions. Deposits with these sorts of structures probably have a relatively longer transport history from eruptive source to depocenter, but likely substantially less than one year (e.g., ref. 7).

S1.1.1c References

1. Miall AD (1996) *The Geology of Fluvial Deposits: sedimentary facies, basin analysis and petroleum geology*. (Springer-Verlag, Berlin).
2. Collinson JD, Thompson DB (1989) *Sedimentary Structures* (2nd edition). (Unwin Hyman, London).
3. Allen JP, Fielding CR (2007) Sedimentology and stratigraphic architecture of the Late Permian Betts Creek Beds, Queensland, Australia. Sed Geol 202:5-34.
4. Harms JC, Southard JB, Spearing DR, Walker RG (1982)*Structures and sequences in clastic rocks. Society of Economic Paleontologists and Mineralogists* Short Course 9, Tulsa.
5. Farrell KM (1987) In: *Recent Developments in Fluvial Sedimentology*. F.G. Ethridge, R.M. Flores, M.D. Harvey, Eds. (Society of Economic Paleontologists and Mineralogists Special Publication 39, Tulsa) pp 111-120.
6. Swisher CC et al. (1996) Latest *Homo erectus*, in Java: potential contemporaneity with *Homo sapiens* in Southeast Asia. Science 274:1870-1874.
7. Mack GH, Macintosh WC, Leede, MR, Monger HC (1996) Plio-pleistocene pumice floods in the ancestral Rio Grande, southern Rio Grande rift, USA Sed Geol 103:1-8

S1.2 Figure Legends

Figure S1.2.1. Sedimentary features of the "20-m terrace". A: gravel lag; note distal metapodial in white circle; scale is 15 cm. B: large-scale planar cross-stratifications in pebbly sands; white arrows denote local tops of individual cross-strata; black arrows at lower contact with marine Kalibeng carbonate; gray bar is 10 cm. C: tuff (bentonite) lens bracketed by coarse sands and gravels; note red pocketknife as 6 cm scale. D: interbedded tuffaceous fine sands, silts, and clays; staff at right is 150 cm.

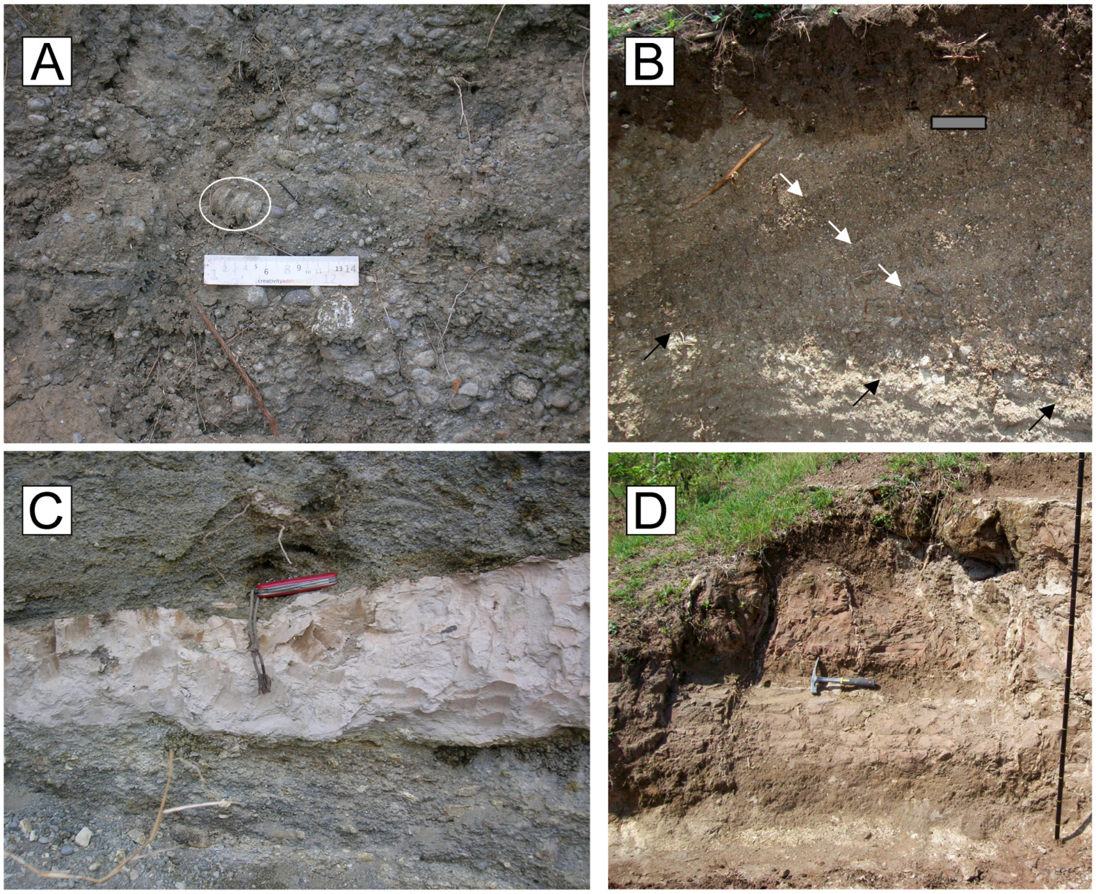
A
B
C
D
