# Supplementary material for: The Age of the 20 Meter Solo River Terrace, Java, Indonesia and the Survival of Homo erectus in Asia
Source: PLoS One. 2011 Jun 29;6(6):e21562. doi: 10.1371/journal.pone.0021562 (PMC3126814; doi:10.1371/journal.pone.0021562)

Appendix S2. **Taphonomy of faunal remains from Jigar**
Supporting Information for The age of the 20 meter Solo River Terrace, Java, Indonesia and the survival of *Homo erectus* in Asia. Etty Indriati, Carl C. Swisher III, Christopher Lepre, Rhonda L. Quinn, Rusyad A. Suriyanto, Agus T. Hascaryo, Rainer Grün, Craig S. Feibel, Briana L. Pobiner, Maxime Aubert, Wendy Lees, Susan C. Antón

S2.1 Taphonomy: Materials, methods, and results

We evaluated the faunal assemblages recovered during the 1970s excavations at Jigar by GMU and those conducted by SoRT in 2006 for taphonomic signals of site formation processes. Over the course of these excavations a total of eleven 2m x 2m squares were excavated, elements piece-plotted, and their preferred orientation recorded. These excavations yielded abundant faunal remains representing a subset of the taxa form Ngandong (1-4). From the 1970's excavations, 187 total bone (n=153) and tooth (n=34) fragments were studied. From the 2006 excavations, we studied 385 bone fragments and 97 tooth fragments. All remains are housed at Gadjah Madah University. Teeth from the 1970s fauna yielded similar U-series and ESR ages as those from Ngandong (5).

Several variables were investigated to address the possibility of input from multiple time periods to the Jigar assemblages. To assess the likelihood of transport/fluvial activity affecting the spatial integrity of each assemblage, we measured the maximum length of each specimen to the nearest centimeter and examined each for rolled or rounded edges and evidence of sedimentary abrasion (n=123 from the 1970s excavations and n= 374 from 2006). We assessed the consistency of the taphonomic signal by assessing weathering, fracture patterns, and matrix type. We scored weathering stage[6], which can indicate the length of time a bone was exposed on the surface before burial. We also recorded evidence of spiral fracture and cortical spalling, as well as expanded matrix distortion (EMD; ref 7). We considered the type of matrix adhering to each specimen. To consider the possibility of carnivore or hominin agency in assemblage composition, we scored evidence of fresh fracture, cut or percussion markings, and carnivore tooth notching (following refs. 8-10)

Assemblage composition: The majority of both assemblages are mammalian remains unidentifiable to skeletal part or taxon [119 (63%) in 1970s; 290 (75%) in 2006]. Amongst the dental remains, most were identifiable as either bovid or cervid teeth [32 (94%) of all teeth of 1970s; 95 (98%) of 2006]. Other vertebrates include turtle (n =3 carapace fragments) and crocodile (n=1 tooth). Fifteen percent (n = 23) of the bones and bone fragments of the 1970s are identifiable to skeletal part. Of these, 14 are cranial bones and 9 are post-cranial (3 axial, 6 appendicular). Of the 385 bone specimens from the SoRT 2006 excavations, ten percent (n = 37) were identifiable to body part (1 cranial fragment and two horn core fragments; 3 cervical and 4 thoracic vertebrae; one rib; parts of 3 femora and 4 tibiae, one humerus, one radius and one ulna; 2 scapulae and 1 innominate; 6 metapodials, 5 carpals/tarsals). An additional 53 bones were identified as long bones (n=43 shafts, n=10 epiphyses).

Fluvial activity, size sorting, rounding/rolling, and abrasion: The assemblages are not well sorted although they were clearly water-lain; cross-bedding was reported from

the 1970s excavations and was evident in the sediments excavated in 2006. No predominant flow direction is evident from the orientation of the specimens themselves, although there is a preferred Northeast orientation of crossbedding in the sediments. The 1970s assemblages yield no evidence of preferential bone size, nor any indication that small bones have been winnowed out of the assemblage (Fig. S2.2.1). Likewise, most bones (76%) from the 1970s excavation exhibit no evidence of rounded or rolled edges; 24% exhibit slight rounding, and only one specimen exhibited moderate rounding. The bones were slightly more rounded and rolled in the 2006 assemblage: 43% exhibited no rounding or rolling, another 43% exhibited slight rounding, 12% exhibited moderate rounding, and 2% exhibited heavy rounding. None of the specimens had sedimentary abrasion marks. We conclude that each assemblage is minimally transported.

Evidence of temporal difference in time to burial. Weathering stage suggests that the bones were buried relatively rapidly (<6 years, mainly <1 year). Of the subset of bones from the 1970s for which weathering stage could be determined, 22 exhibited no weathering, 4 exhibited stage 1 weathering, and 2 exhibited stage 2 weathering. The 2006 assemblage exhibits a similar pattern: of the 24 bones for which weathering stage could be confidently determined, 20 (83%) exhibited no weathering, three exhibited stage 1 weathering, and 1 exhibited stage 2 weathering. Some of the bone specimens from the 1970s (n=15) exhibited major cortical bone exfoliation, while a few (n=4) exhibited surficial pitting from some corrosive process. A higher proportion of the bones from the 2006 excavation exhibited exfoliation (n=40) and corrosion (n=72). Many of the bones from the 1970s excavation also exhibit "expanded matrix distortion" (EMD; 7), where matrix infilled the cracks of a bone pre-fossilization, causing expansion cracking. There is also matrix adhering to the outer surface of many of the bones, which along with other surface-obscuring taphonomic processes renders the cortical surface of about half of the Jigar bones "unreadable" for bone surface modifications (Fig. S2.2.2). The actual matrix adhering to the bones varies slightly from one specimen to another but has an overall high level of uniformity on an assemblage-wide scale and across the two excavations.

Hominin or carnivore agency. There is no evidence for fresh (green) breakage on any of the 1970s Jigar specimens, although it is possible that matrix adhering to the bones is obscuring the tell-tale spiral fracture morphology. Forty three of the 2006 Jigar specimens exhibit spiral breaks, which may be indicative of fresh breakage. Thirty one (25%) of the 1970s specimens and 124 (35%) of the 2006 specimens had modern breaks; in the 1970s assemblage, some of these breaks were recognized previously and glued during excavation or curation. There is also no evidence of cut or percussion marking, nor any associated stone tools or other cultural material that would indicate unequivocal evidence of hominin involvement. There is one larger mammal long bone midshaft fragment with a possible carnivore notch (9).

Conclusions

We found evidence of a short time to burial and no evidence of multiple assemblages. We conclude that the bias towards larger mammals in the assemblages likely reflects real patterns in the paleoenvironment, and not taphonomic factors related to either mixed temporal assemblages or high energy flow. The Jigar assemblage does

not appear to represent primarily either a lag deposit, as has been suggested for the Omo collections (cf. ref.11), or an entirely transported deposit resulting from a hydraulically sorted, high energy flow. It more likely represents a low energy alluvial environment representing either a single or successive high water (flood) levels. If evidence from the assemblage from Jigar can be extrapolated to the case at Ngandong, then the absence of evidence for significant transport of elements and the absence of multiple assemblages strengthens the argument against preferentially reworked assemblages there.

References

1. Nitihaminoto and Moelyadi. (1980). Report on excavation at Jigar, Blora Regency, Central Java. (Translated from Indonesian) Proy Pen Paleoanthro Nas Yogyakarta.
2. Moelyadi I (1977) Geological report on terrace sediment in the Ngandong region, Central Java (Translated from Indonesian). Proy Pen Paleoanthro Nas Yogyakarta.
3. Moelyadi I (1980) Research on excavation of the terrace sediment in Ngandong V, Blora Regency, Central Java (Translated from Indonesian). Proy Pen Paleoanthro Nas Yogyakarta.
4. Indriati E, Pobiner B, Antón SC (2006) Taphonomy and site formation of the 20m Solo River Terrace, Java Indonesia: Implications of Late Pleistocene *H. erectus* Am J Phys Anth S42**:**107-108 (2006).
5. Swisher CC III *et al.* (1996) Latest *Homo erectus*, in Java: potential contemporaneity with *Homo sapiens* in Southeast Asia. Science 274:1870-1874.
6. Behrensmeyer AK (1978) Taphonomic and ecologic information from bone weathering. Paleobiology 4:150-162.
7. White T (2003) Early Hominids--Diversity or Distortion? Science 299:1994-1997.
8. Blumenschine RJ, Marean CW, Capaldo SD (1996) Blind Tests of Inter-analyst Correspondence and Accuracy in the Identification of Cut Marks, Percussion Marks, and Carnivore Tooth Marks on Bone Surfaces J. Archaeol Sci. 23**:**493-507.
9. Capaldo S,Blumenschine RJ (1994) A quantitative diagnosis of notches made by hammerstone percussion and carnivore gnawing on bovid long bones. American Antiquity 59:724-748.
10. Lyman L (1994) *Vertebrate Taphonomy* (Cambridge Univ. Press, Cambridge).
11. Boaz N, Behrensmeyer AK (1976) Hominid taphonomy: Transport of human skeletal parts in an artificial fluviatile environment. Am J Phys Anth 45:53-60.

S2.2 Figure Legends for Taphonomy

Figure S2.2.1: Maximum Bone Length for: a) 1970s assemblage and b) 2006 assemblage.

Figure S2.2.2: Surface readability of: a) 1970s assemblage and b) 2006 assemblage.

Figure 2.1.1a

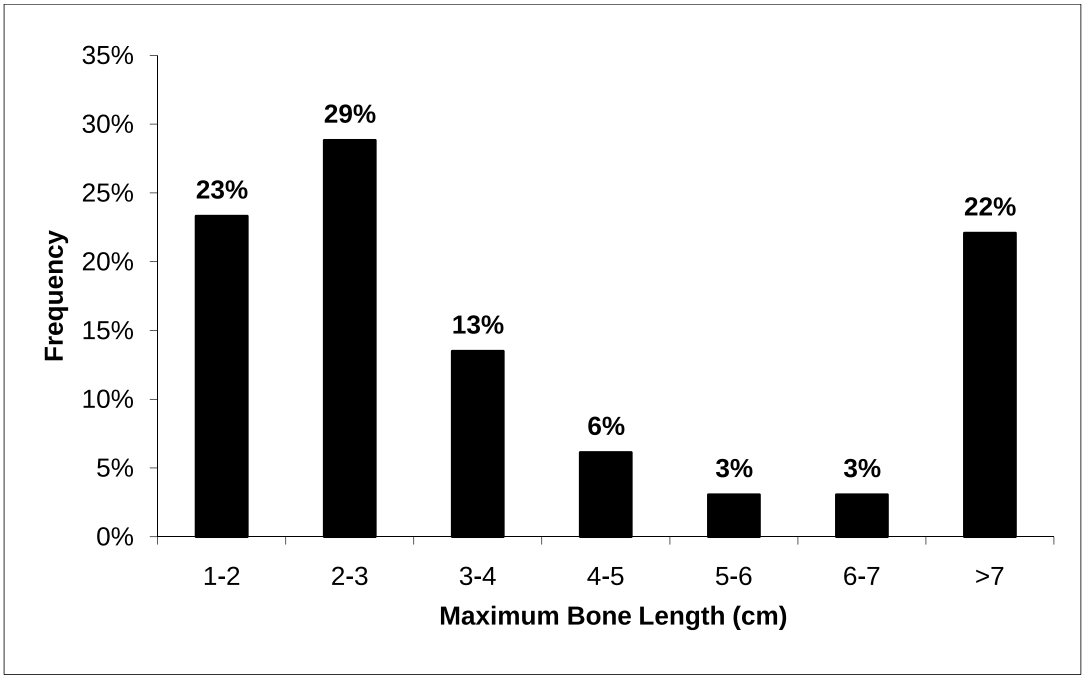

Figure 2.1.1b

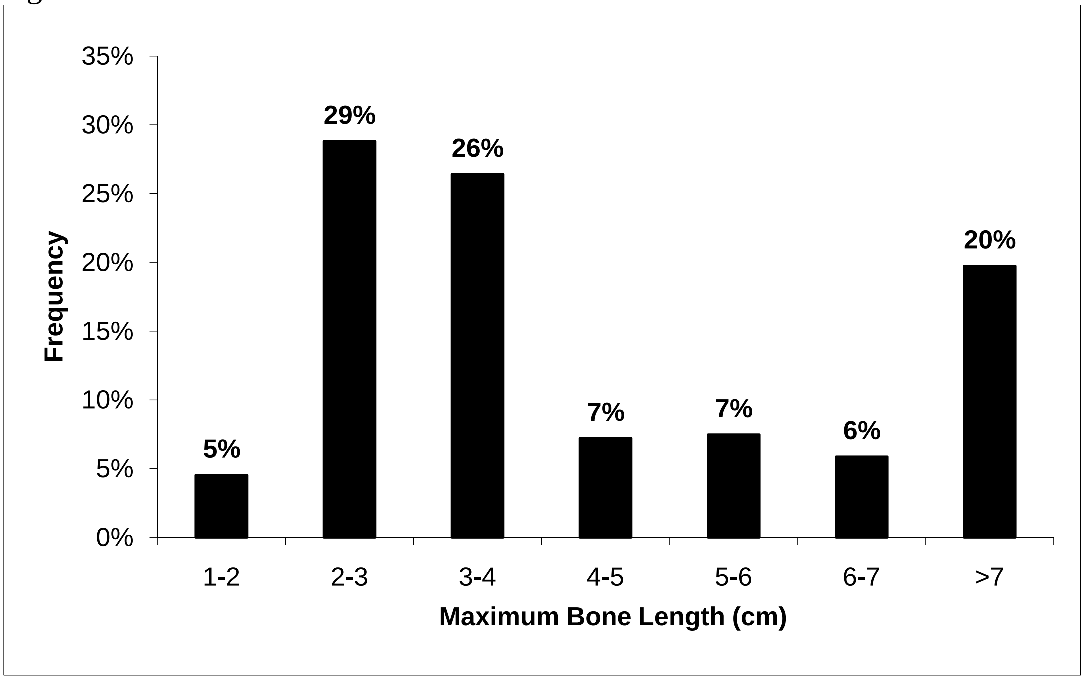

Figure 2.1.2a

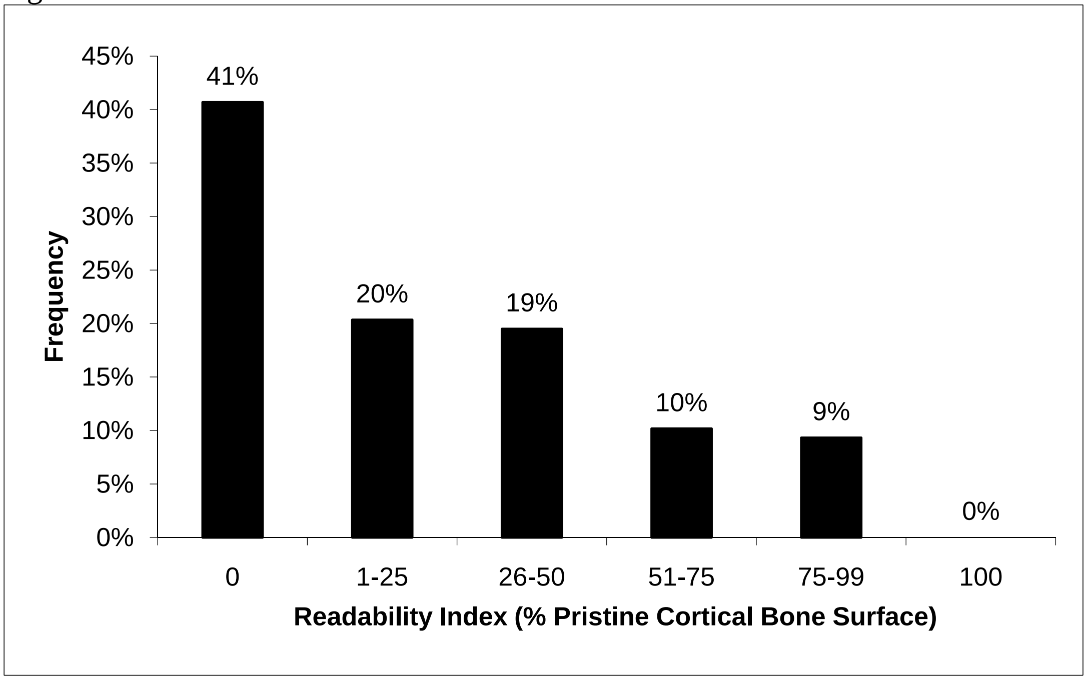

Figure 2.1.2b

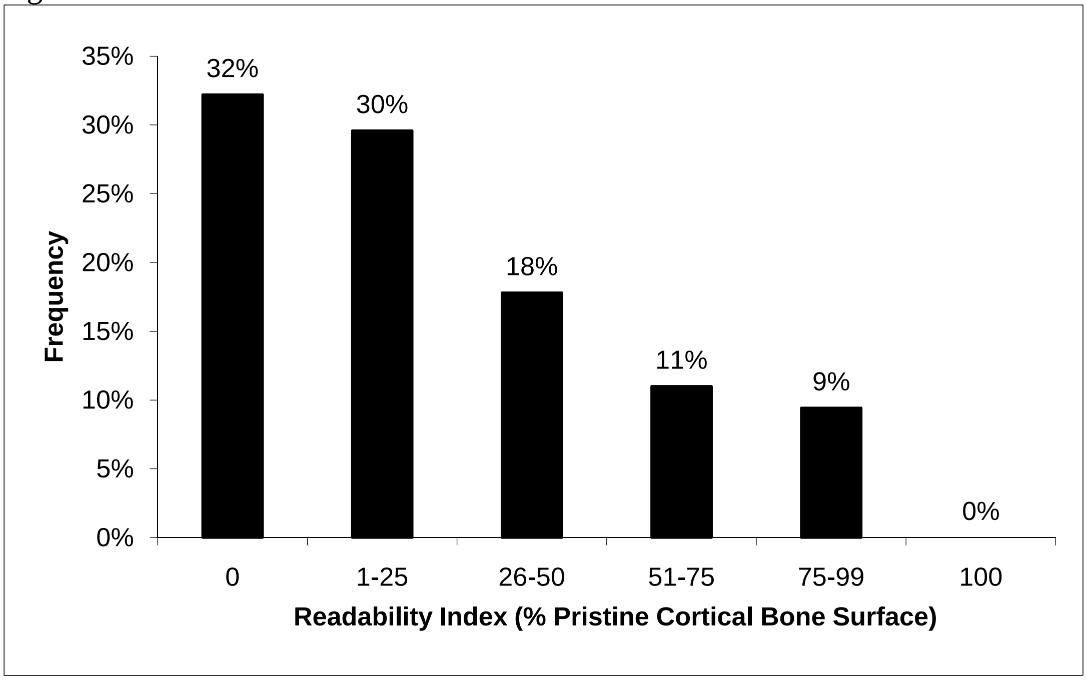

Supplement: Appendix S2 — Taphonomy of faunal remains from Jigar. 2.1 Taphonomy: materials, methods, and results. 2.2 Figures and legend for taphonomy. Figure 2.2.1 Maximum bone length. Figure 2.2.2 Surface readability. (PDF) [file pone.0021562.s004.pdf]
